# Glucocorticoid Receptor Signaling in NSCLC: Mechanistic Aspects and Therapeutic Perspectives

**DOI:** 10.3390/biom13091286

**Published:** 2023-08-23

**Authors:** Kostas A. Papavassiliou, Nektarios Anagnostopoulos, Athanasios G. Papavassiliou

**Affiliations:** 1 First Department of Respiratory Medicine, ‘Sotiria’ Hospital, Medical School, National and Kapodistrian University of Athens, 11527 Athens, Greece; konpapav@med.uoa.gr (K.A.P.); aris.anag@yahoo.gr (N.A.); 2 Department of Biological Chemistry, Medical School, National and Kapodistrian University of Athens, 11527 Athens, Greece

**Keywords:** non-small cell lung cancer, glucocorticoid receptor, glucocorticoids

## Abstract

Recent advances in non-small cell lung cancer (NSCLC) biology and the discovery of novel therapeutic targets have led to the development of new pharmacological agents that may improve the clinical outcome of patients with NSCLC. The glucocorticoid receptor (GR) is an evolutionarily conserved protein belonging to the nuclear receptor superfamily of transcription factors and mediates the diverse actions of glucocorticoids in cells. Data suggest that the GR may play a relevant role in the molecular mechanisms of NSCLC tumorigenesis and malignant progression. Additionally, evidence indicates that glucocorticoids may affect the efficacy of standard treatment, including chemotherapy, immune checkpoint inhibitors, and targeted therapy. Furthermore, several findings show that GR expression may probably be associated with NSCLC patient survival. Finally, glucocorticoids may be used as therapeutic agents for the clinical management of NSCLC patients. Here, we briefly review the latest advances on the biological role of GR signaling in NSCLC and discuss the potential use of the GR as a prognostic and predictive biomarker. Importantly, we explore the therapeutic potential of glucocorticoids and the effect of adding such drugs to standard therapies for NSCLC.

## 1. Introduction

Lung cancer is the second most commonly diagnosed cancer worldwide, following breast cancer, and its incidence is still growing. Advances in the field of lung cancer biology have allowed for drastic changes in recent years regarding the treatment landscape of lung cancer. Nevertheless, lung cancer remains by far the leading cause of cancer deaths, surpassing each year the number of people dying from colon, breast, and prostate cancers combined [1]. Non-small cell lung cancer (NSCLC) represents the most common type of lung cancer, accounting for approximately 80% to 85% of all lung cancer cases. Surgery, along with chemoradiotherapy, have long formed the backbone of NSCLC treatment. However, the majority of patients develop therapy resistance and eventually relapse [2]. New therapeutic strategies have been explored and the introduction of small molecule tyrosine kinase inhibitors and immune checkpoint inhibitors has resulted in unprecedented survival benefits in patients harboring particular genetic alterations or expressing tumor immunity-related biomarkers. Tyrosine kinase inhibitors have paved the way towards a targeted therapeutic approach based on the molecular profile of NSCLC patients and new potential players driving NSCLC development and progression are continuously emerging. The glucocorticoid receptor (GR) belongs to the nuclear hormone receptor superfamily and functions as a ligand-activated transcription factor to maintain cellular homeostasis. In addition to its essential role in human physiology, the GR is involved in the pathophysiology of several diseases, including cancer [3,4]. Specifically, there is a substantial amount of evidence suggesting the involvement of GR signaling in NSCLC biology [5]. In the present article, we highlight the molecular underpinnings of GR signaling and probe the clinical potential of the GR and glucocorticoids in the setting of NSCLC.

## 2. Structure and Function of the GR

The *nuclear receptor subfamily 3 group C member 1* (*NR3C1*) gene, which is found on chromosome 5 (5q31.3) and is composed of 10 exons (1 to 9 beta), encodes the GR. The GR protein is a multidomain transcription factor organized into three major functional domains, including the N-terminal domain (encoded by exon 2), the C-terminal ligand-binding domain (encoded by exons 5 to 9), and the DNA-binding domain (encoded by exons 3 and 4) [6]. When the GR does not bind to its ligands, namely glucocorticoids, it is localized in the cytoplasm where it physically interacts with a macromolecular complex consisting of chaperones and other proteins. The binding of glucocorticoids to the GR triggers hyperphosphorylation, which results in conformational changes in the protein that subsequently lead to the detachment of the GR from the inactive, cytoplasmic multiprotein complex. In its hyperphosphorylated active state, the receptor forms homodimers and translocates to the nucleus, where it can act through several ways: (a) Binding in a direct manner to DNA alone or in association with other transcription factors to regulate gene transcription, or (b) Binding to other DNA-bound transcription factors. Additionally, GR signaling acts via non-genomic and epigenetic mechanisms to upregulate or downregulate the expression of a plethora of target genes. GR signaling is well-known for its anti-inflammatory effects, whereby the glucocorticoid-bound GR enters the nucleus and physically interacts with the transcription factors nuclear factor-κB (NF-κB) or activator protein-1 (AP-1), repressing the transcription of pro-inflammatory genes [7]. The effects of GR signaling are context dependent and this specificity is well-reflected in the respiratory system where it is involved in lung development by promoting the maturation and surfactant protein expression and secretion by epithelial lung cells [8,9,10].

## 3. Biological Role of GR Signaling in NSCLC Biology

GR signaling has been associated with various aspects of cancer development and progression. Preclinical studies on cancer biology have revealed a multifaceted role of the GR that depends on the cancer cell type behaving either as a tumor suppressor or an oncogene [4]. So far, GR signaling has been implicated in the biology of various hematological malignancies as well as solid cancers including lung cancer, prostate cancer, breast cancer, pancreatic cancer, ovarian cancer, endometrial cancer, bladder cancer, colon cancer, liver cancer, and brain cancer [4]. Importantly, there is evidence that treatment with GR ligands affects the role of the GR, altering its function.

**Figure 1:**
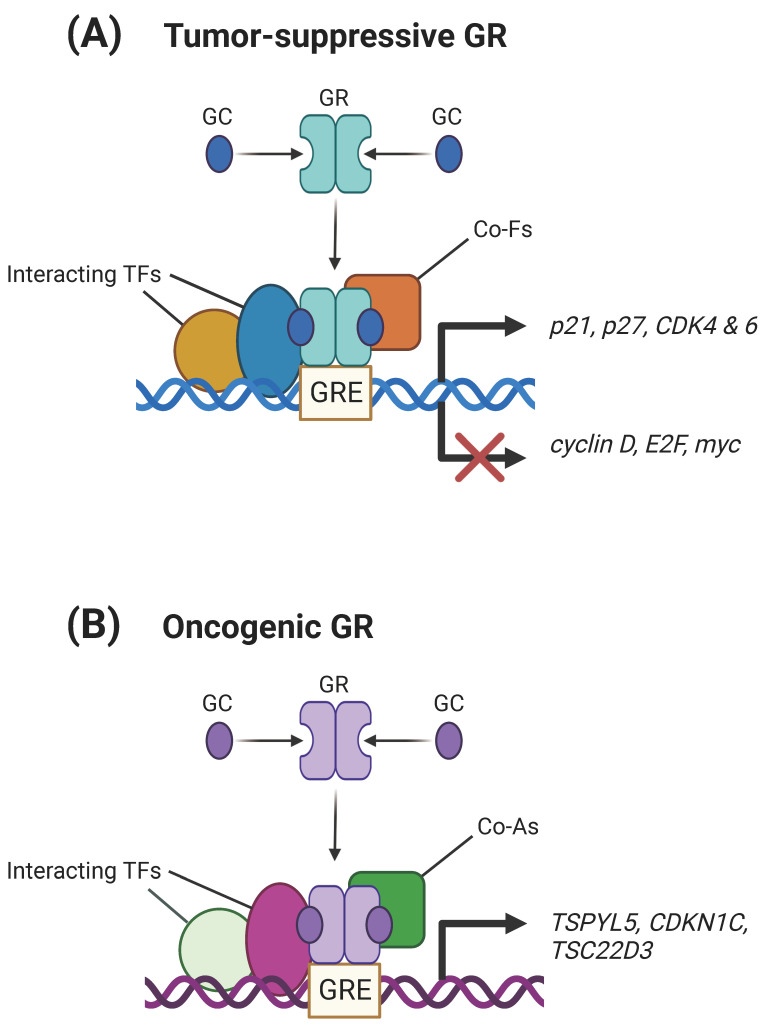
The role of the GR in NSCLC biology. (**A**) The tumor-suppressive properties of the GR include the upregulation of genes involved in cell cycle arrest (e.g., *p21* and *p27*) and the downregulation of genes associated with cell cycle progression (e.g., *cyclin D*) [11,12]. (**B**) The oncogenic functions of the GR include the induction of genes that promote cancer cell growth and migration (e.g., *TSPYL5*) [17]. Co-As, co-activators; Co-Fs, co-factors (co-activators or co-repressors); GC, glucocorticoid; GR, glucocorticoid receptor; GRE, glucocorticoid response element; TFs, transcription factors. This figure was created using the tools provided by BioRender.com (accessed on 17 August 2023).

In the context of NSCLC, the majority of data points toward a tumor-suppressive role of the GR (Figure 1). Greenberg et al. showed that dexamethasone, a synthetic glucocorticoid that binds to the GR, suppresses the proliferation of A549 lung adenocarcinoma cells by inducing cell cycle arrest. The authors found that this effect was a result of retinoblastoma (Rb) protein hypophosphorylation, decreased activity of the cyclin-dependent kinases 2 and 4 (CDK2/4), decreased protein levels of cyclin D, E2 transcription factor (E2F) and Myc, and increased protein levels of the CDK inhibitor p21. Furthermore, dexamethasone downregulated the activity of the extracellular signal-related kinase (ERK)/mitogen-activated protein kinase (MAPK) signaling pathway and additional data suggested that this may be responsible for the observed cell cycle arrest and growth inhibition of lung adenocarcinoma cells. All of these effects were demonstrated to be mediated by the GR [11]. In another study, Srivastava et al. showed that treatment of A549 cells with dexamethasone caused a reduction in cancer cell migration, invasion, colony-forming ability, and adhesion, the latter as a result of damaging cortical actin. Additionally, dexamethasone treatment led to cell cycle arrest of A549, which is mediated through the upregulation of the protein expression of CDK inhibitors p21 and p27, together with that of CDKs 4 and 6. Dexamethasone also induced the hyperphosphorylation of the Rb protein, resulting in the irreversible senescence of A549 cells. Using clinical datasets, the authors found that GR expression was decreased in NSCLC patients, while a higher expression was associated with a better overall survival [12]. In agreement with this, Surati et al. found that the overexpression of the GR was correlated with improved overall survival through the use of a comprehensive translational thoracic oncology database (the Thoracic Oncology Program Database Project) [13]. Likewise, Lu et al. used immunohistochemistry to support high protein levels of the GR being present in approximately half of advanced NSCLC patients and that this increased GR expression is related to a better clinical outcome [14]. In a similar fashion, Srivastava et al. analyzed clinical datasets and observed that an increased expression of both the GR and c-Jun N-terminal kinase (JNK) was associated with a favorable prognosis. The authors also provide data on the molecular basis of this correlation, revealing that the treatment of NSCLC cells with dexamethasone triggers the phosphorylation of JNK, which interacts with the GR and hinders its ubiquitin-mediated proteasome degradation [15]. In a recent study, Matthews et al. identified a novel non-genomic tumor-suppressive role for the GR in mitosis. Using a series of elegant experiments, the authors discovered that the GR accumulates at the spindle centrosome during mitosis where it is phosphorylated by the Aurora A kinase, a centrosome-associated protein essential for the progression of mitosis. The knockdown of the *GR* led to a delay in mitosis, frequent mitotic aberrations, and cell death. Mice harboring only one *GR* gene copy displayed increased numbers of tumors and further genetic loss of the *GR* locus. Altogether, these data indicate that the GR regulates accurate chromosome segregation and that a decrease or loss in its expression results in malignant behavior [16].

Notwithstanding, there are a few studies which lend an oncogenic role to the GR (Figure 1). A recent study by Sasaki et al. identified the GR/serum and glucocorticoid-induced protein kinase 1 (SGK-1)/N-myc downstream-regulated gene 1 (NDRG-1) pathway as a positive predictor of recurrence and a poor prognostic factor in lung adenocarcinoma [18]. Moreover, in a different study, Lakshmanan et al. presented findings that indicated that the mucin 16 (MUC16)/Janus kinase 2 (JAK2)/signal transducer and activator of transcription 3 (STAT3)/GR signaling axis promotes NSCLC. In particular, the authors showed that the MUC16-mediated phosphorylation of both JAK2 and STAT3 results in STAT3 nuclear translocation, where it binds to the GR. In turn, the STAT3–GR complex binds to the glucocorticoid response element (GRE)-containing promoter of the *testis-specific Y-like protein 5* (*TSPYL5*) gene, upregulating its expression and, subsequently, promoting NSCLC cell growth and migration [17]. Another study in favor of an oncogenic role for the GR demonstrates that GR signaling causes cancer cells to acquire a reversible dormant state which is characterized by a resistance to multiple anticancer drugs and an upregulation of insulin-like growth factor 1 receptor survival signaling vulnerability to inhibitors. The authors of this study provide data showing that this cancer cell dormancy phenotype is due to the GR-induced expression of its target gene *cyclin-dependent kinase inhibitor 1C* (*CDKN1C*) through long-distance chromatin interactions [19].

## 4. Clinical Potential of GR and Glucocorticoids for NSCLC Patients

It is clear from the above that the GR plays a role in the development and progression of NSCLC. Most data hint toward a tumor-suppressive role of the GR in NSCLC; however, we cannot ignore the studies that suggest its oncogenic features when considering the use of glucocorticoids in cancer patients. An important aspect of GR signaling that is clinically relevant is its targeting of glucocorticoids. Interestingly, the GR-mediated effects of glucocorticoids are not straightforward, and it appears that treatment with glucocorticoids can alter the function of the GR in particular contexts. Early on, Hofmann et al. had shown that dexamethasone has growth-inhibitory effects in several human NSCLC cell lines, especially those expressing high levels of the GR [20]. Along the same lines, Liang et al. found that budesonide, a GR agonist, inhibited the proliferation of TP53 wild-type A549 cells. This anti-proliferative effect is associated with cancer cell cycle arrest and might be mediated, in part, by the suppression of ERK and NF-κB signaling [21]. Huffman et al. discovered that the treatment of serine/threonine kinase 11/liver kinase B1 (STK11/LKB1) mutant NSCLC cells with dexamethasone led to cell cycle arrest via the GR-mediated activation of *CDKN1C* expression. The presence of the STK11 mutation, along with increased expression of the carbamoyl phosphate synthase 1 (CPS1) protein, were identified as predictive biomarkers of this therapeutic vulnerability [22]. Clobetasol propionate, a synthetic glucocorticoid, was used by Choi et al. to suppress the in vitro and in vivo growth of Kelch-like ECH-associated protein 1 (KEAP1) mutant NSCLC. In terms of mechanism, the authors found that clobetasol propionate, acting via the GR, prevented the accumulation of the nuclear factor E2-related factor 2 protein within the nucleus and promoted the latter’s degradation [23]. Evidence also suggests that glucocorticoids are able to affect the efficacy of standard treatments used in the clinical management of NSCLC patients. A recent study by Parajuli et al. uncovered a novel strategy to enhance the efficacy of immunotherapy in NSCLC. Specifically, the pretreatment of lung adenocarcinoma cells with dexamethasone induces a cancer cell senescence that results in enhanced infiltration of the tumor with T and natural killer cells. This immunomodulatory effect may improve the anticancer activity of currently used immunotherapies [24].

On the other hand, Cui et al. recently reported that increased endogenous glucocorticoid levels were associated with decreased immunosurveillance and a poor response to immune checkpoint inhibitors [25]. Similarly, Yang et al. revealed that a stress-induced rise in plasma corticosterone and the upregulation of the expression of TSC22 domain family protein 3 (TSC22D3), a glucocorticoid-induced transcriptional regulator with immunosuppressive effects, blocks type I interferon responses in dendritic cells as well as the activation of T cells. The end result is a compromise in therapy-related antitumor immunity [26]. Several studies show that the use of glucocorticoids in cancer may have negative implications for cancer patients. For example, Caratti et al. found that glucocorticoids intercept the ability of the GR to inhibit cell proliferation and tumor growth through the Ras signaling blockade. In particular, the authors’ data demonstrated that when the GR is not ligand-bound, it is located in the cytoplasm in association with KRAS-containing protein complexes, thus inhibiting oncogenic signaling [27]. Jeon et al. discovered that dexamethasone inhibits the apoptotic process triggered by the tumor necrosis factor (TNF)-related apoptosis-inducing ligand via the glycogen synthase kinase-3beta (GSK-3β)-mediated downregulation of death receptor 5 and the upregulation of cellular FLICE-inhibitory protein (FLIP) in A549 lung adenocarcinoma cells [28]. Moreover, research hints at the fact that glucocorticoids interfere with the anticancer effects of chemotherapy. Mechanistically, data show that glucocorticoids induce resistance to apoptosis, suppress the fraction of cancer cells found in the chemotherapy-sensitive S-phase, and decrease the gene transcription of drug transporters, especially in cancer cells with high GR expression [29,30]. Interestingly, GR expression may function as a predictive biomarker for pemetrexed efficacy in advanced NSCLC patients [31]. A recent study used radiolabeled 3′-Deoxy-3′-^18^F-fluorothymidine with positron emission tomography (PET) to noninvasively monitor the S-phase suppression mediated by glucocorticoids in NSCLC. This useful method is a means to personalize chemotherapy in NSCLC patients under pemetrexed [32]. The negative effect of glucocorticoids probably applies to targeted therapies as well. Su et al. conducted a retrospective cohort study using data from a hospital’s database and found that epidermal growth factor receptor (EGFR)-tyrosine kinase inhibitor (TKI)-treated patients who also received concurrent therapy with glucocorticoids had a higher risk of treatment failure compared with patients treated only with EGFR-TKIs [33].

## 5. Conclusions

As is the case for other solid tumor types, e.g., breast cancer [34], GR signaling is indubitably implicated in the biology of NSCLC. However, there are many questions that need to be answered regarding its function. Given the fact that in the last four decades clinicians have administered synthetic glucocorticoids such as dexamethasone and prednisone, along with chemotherapy to relieve the latter’s side effects and make it tolerable to cancer patients, it is of paramount importance to elucidate their GR-mediated effect on cancer cells and to clarify whether their co-administration with chemotherapy is safe and effective. There is strong evidence that the GR contributes to cisplatin resistance in various solid cancers, including NSCLC, and one of its underlying mechanisms has already been revealed [35,36]. Delving deeper into the GR-dependent drug resistance mechanisms and developing therapeutic strategies to counteract them will allow clinical oncologists to safely and effectively continue using synthetic glucocorticoids to alleviate chemotherapy-induced side effects, as well as improving the clinical outcome of NSCLC patients receiving chemotherapy together with glucocorticoids.

Through our present work, it becomes obvious that the GR still holds a conflicting role in NSCLC cells, either acting as a tumor-suppressor or an oncogene, depending on the molecular profile (Figure 1). Therefore, further research is required in order to dissect the exact role of the GR in the development and progression of NSCLC. Importantly, researchers should also delineate how GR signaling affects the efficacy of targeted therapy and immunotherapy. Specifically, whether the GR affects the expression of immune checkpoint inhibitors in NSCLC should be evaluated, since there is already evidence that GR signaling regulates PD-L1 expression and, thus, promotes immune evasion and immunotherapy resistance in other cancers [37,38,39]. Finally, future studies should probe and validate the potential use of the GR—possibly coupled with other cancer-associated steroid hormone nuclear receptors, e.g., androgen receptor (AR; NR3C4) [40,41]—as a prognostic and predictive biomarker in NSCLC.
